# Bacterial cellulose as an example product for sustainable production and consumption

**DOI:** 10.1111/1751-7915.12744

**Published:** 2017-07-11

**Authors:** Woo Dae Jang, Ji Hyeon Hwang, Hyun Uk Kim, Jae Yong Ryu, Sang Yup Lee

**Affiliations:** ^1^ Metabolic and Biomolecular Engineering National Research Laboratory Department of Chemical and Biomolecular Engineering (BK21 Plus Program) Institute for the BioCentury Korea Advanced Institute of Science and Technology (KAIST) Daejeon 34141 Republic of Korea; ^2^ BioInformatics Research Center KAIST Daejeon 34141 Republic of Korea; ^3^ BioProcess Engineering Research Center KAIST Daejeon 34141 Republic of Korea

## Abstract

Life cycle of bacterial cellulose. Sustainable production and consumption of bio‐based products are showcased using bacterial cellulose as an example.

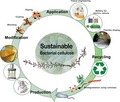

Sustainable development has become one of the most important agendas for microbial metabolic engineers. What is sustainable development? Modern concept of sustainable development was first defined in the Brundtland Report published by the United Nations World Commission on Environment and Development back in 1987 (Brundtland, 1987). Purpose of the report was to seek a solution that enables both environment protection and economic growth rather than compromising each other. The definition of sustainable development has continued to evolve thereafter through international efforts, for example, Agenda 21 in 1992 and Millennium Development Goals (MDGs) in 2000, both organized by the United Nations. In September 2015, the United Nations General Assembly formally accepted a new set of seventeen measurable Sustainable Development Goals (SDGs), ranging from ending world poverty and hunger to combating climate change by 2030 (https://sustainabledevelopment.un.org/).

Microbial metabolic engineers have continued to strive to establish sustainable biosystems for the production of industrially useful chemicals and materials from renewable non‐food biomass. In this regard, the mission of metabolic engineering aligns well with SDGs, especially SDG 12 that states to ‘ensure sustainable consumption and production patterns’. Microbial fermentative production of chemicals and materials from renewable resources can contribute to SDG 12 both environmentally and economically. Systems metabolic engineering, that integrates metabolic engineering with systems biology, synthetic biology and evolutionary engineering, has allowed efficient bio‐based production of increasing number of chemicals and materials (Lee *et al*., 2012; Choi *et al*., 2015, 2016; Lee and Kim, 2015). Among these products, we will use bacterial cellulose (BC) as an example product to establish sustainable production and consumption. BC has a huge potential to reshape our daily lives and industry (Fig. 1). Because contribution of BC to environment through its biodegradability is quite obvious (e.g. by using cellulase), more emphasis will be given on new economic opportunities through various industrial applications of BC in this article.

**Figure 1:**
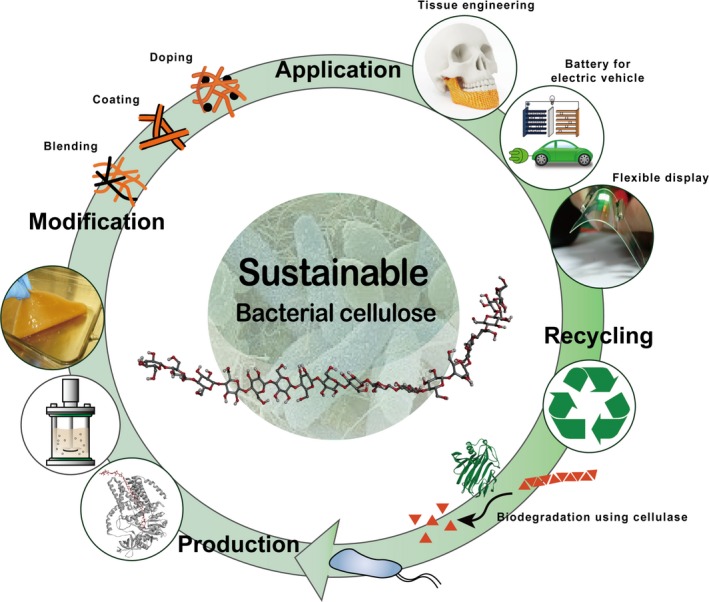
Life cycle of bacterial cellulose from microbial production to downstream modifications, applications and biodegradation. Bacterial cellulose with the life cycle shown here clearly demonstrates how bio‐based production of chemicals and materials helps us move towards sustainable production and consumption.

Cellulose is one of the most widely used natural materials and has traditionally been used for paper and textiles in addition to forest products. While cellulose is a well‐known plant product, some bacteria have gained attention as an alternative and sustainable source of cellulose with *Komagataeibacter xylinus* being a representative BC producer. BC and plant cellulose have the same molecular formula, but the former has additional competitive advantages that have attracted material scientists’ attention. In comparison with plant cellulose, BC has excellent physical properties with respect to mechanical stability, tensile strength, thermostability, crystallinity (i.e. ultrafine fibrous structure), purity and biocompatibility (i.e. biologically non‐toxic and no immune responses) (Barud *et al*., 2011; Cacicedo *et al*., 2016). Although not fully commercialized yet, excellent biocompatibility of BC has motivated development of BC‐based biomedical products such as bone tissue scaffolds, artificial blood vessels, artificial skin and dental implants (Petersen and Gatenholm, 2011). Adhesion of cells to the surface of BC is critical in tissue engineering and artificial scaffolds. Therefore, various molecules promoting cell adhesion have been attempted such as arginine–glycine–aspartic acid (RGD) peptides (Andrade *et al*., 2013) or other natural/synthetic polymers possessing better biocompatibility such as gelatin (Kim *et al*., 2010), poly(ethylene glycol) (Cai and Kim, 2010) and collagen (Zhijiang and Guang, 2011). The high purity BC obtainable through fermentation also facilitates separation and purification, and further physical/chemical modifications (Huang *et al*., 2014). With such competitive advantages, application scope of BC has been expanded from food industry to cosmetics, biomedical and even electronics industries. Some of well‐known commercialized BC‐based products include jelly‐like dessert *Nata de coco* originating in the Philippines (Budhiono *et al*., 1999), wound dressing (Czaja *et al*., 2006), facial mask (Ullah *et al*., 2016) and acoustic diaphragm of speakers and headphones (Iguchi *et al*., 2000). For wound dressing, antimicrobial properties can be endowed by incorporating inorganic nanoparticles (Wu *et al*., 2014), antimicrobial peptides (Basmaji *et al*., 2014) and polymers (Figueiredo *et al*., 2013) into BC.

Interestingly, BC is reaching industries that are not seemingly related to biotechnology, such as battery and display industries. Development of electric vehicles and portable electric devices has triggered an increasing demand on energy storage. Battery separator in electrochemical energy storage devices, such as Li‐ion battery, is an excellent example where BC can be deployed, thanks to its high tensile strength, high thermostability and high crystallinity (Zhang *et al*., 2015). Battery separator has to be physically and chemically stable to prevent direct contact between anode and cathode, but should allow efficient transport of ions. Its structure and properties highly influence the battery performance. Because BC possesses unique fibrous and cross‐linked three‐dimensional network structure, it is considered to be an outstanding candidate for next‐generation battery separator. Battery separator made of BC shows thermostability up to 180 °C and good ionic conductivity and is expected to give competitive battery performance (Jiang *et al*., 2015). BC has also been considered as an outstanding ingredient material for a matrix used in flexible optoelectronic films such as organic light‐emitting diodes (OLED) (Legnani *et al*., 2008). BC with high crystallinity, sufficient porosity and high surface area is considered ideal to serve as a matrix to support various functionalized materials (Chen *et al*., 2016). However, transparency and conductivity of BC‐based matrix further need to be improved through modification processes such as acetylation of BC (Ifuku *et al*., 2007) and incorporation of polycaprolactone into BC (Barud *et al*., 2013) (Fig. 1). In the near future, application of optically transparent BC‐based conductive composites will further be extended to a breakthrough transparent display technology such as see‐through display that displays or emits information from itself (e.g. augmented reality), smart glasses that change light transmission in response to altered voltage and wearable display devices. Thus, our current reliance on non‐renewable chemicals and materials for manufacturing these consumer products will decrease by using environmentally friendly and sustainable BC.

An imminent, common challenge with BC for the aforementioned applications is low titre, yield and productivity of BC achievable today. This classical problem can be solved through metabolic engineering and bioprocess optimization. As BC production retards cell growth, it is essential to grow cells to a sufficiently high density before BC accumulation. In the case of *K. xylinus*, a major problem in using glucose as a carbon source is the formation of by‐products including gluconic acid that decrease medium pH, which inhibits BC production (Masaoka *et al*., 1993). Several options to tackle this problem would be to engineer metabolic network to reduce gluconic acid production, optimize glucose feeding strategy and/or to use alternative carbon sources that do not trigger production of by‐products. *K. xylinus* has been observed to utilize rather diverse carbon sources such as arabinose, fructose, glycerol, lactose, malic acid, maltose, mannitol, mannose, methanol and sucrose (Ruka *et al*., 2014). Economic feasibility and renewability can be additionally considered when choosing these alternative carbon sources. Smart use of an additional carbon source can also improve reproducibility that is often a problem unique to microbial biopolymer production. For example, addition of acetic acid was reported to improve the reproducibility of BC production (Toda *et al*., 1997). Further fermentation experiments are needed based upon systematically designed medium composition to boost the BC production in the industrially relevant fermentation setting.

As BC is a secreted high molecular weight polymeric product, the type of a bioreactor influences BC production significantly. Novel bioreactors have been designed specifically for improved BC production while reducing the chance of genetic mutations generating non‐producer cells. For example, rotary and aerosol bioreactors can be employed for better transfer of air and medium nutrients, resulting in better cell growth and BC production. Also, membrane bioreactor allowing lower shear force through static cultivation can be used for higher level BC production than agitated cultivation with greater shear force (Lee *et al*., 2014). Fermentation of *K. xylinus* using aerosol bioreactor has so far yielded the highest BC productivity of 0.38 g l^−1^ h^−1^ among these bioreactors (Lee *et al*., 2014). However, it should be noted that bioreactor type should be carefully determined, depending on desired physical properties of BC to be produced (Campano *et al*., 2016).

Despite a long history of studies on BC and its producer *K. xylinus*, tracing back to 1886 (Brown, 1886), the detailed molecular mechanism of BC biosynthesis only recently started to be elucidated. BC is produced by a membrane‐bound cellulose synthase complex, which synthesizes cellulose from uridine diphosphoglucose (UDP‐glucose). Among subunits constituting the cellulose synthase complex, a conserved catalytic and regulatory subunit BcsA plays the most important role; this subunit is called CesA in plant cells. BcsA consists of glycosyltransferase domain and regulatory PilZ domain (McNamara *et al*., 2015). Glycosyltransferase domain converts α‐form donor glucose from UDP‐glucose into β‐form glucose, which gets attached to BC being extended. The PilZ domain binds to cyclic diguanylic acid (c‐di‐GMP), an allosteric activator, which causes structural change of BcsA and subsequently enhances the activity of cellulose synthase (Ross *et al*., 1987). The crystal structure of cellulose synthase from another BC‐producing bacterium *Rhodobacter sphaeroides* has recently been elucidated, and, also, the catalytic mechanism of glycosyl transfers based on the structural information of cellulose synthase has been revealed (Morgan *et al*., 2013, 2016). Upon transfer of glucose from UDP‐glucose to BC, the extended BC gets translocated through transmembrane pore. However, biochemical driving force of BC translocation has not yet been elucidated. With insights increasingly gained on cellulose synthase, it should be possible to systematically engineer this enzyme complex to improve the polymerization and translocation efficiency.

Despite all the great physical properties of BC and a long history of studies on BC biosynthesis, low BC production performance is the currently the biggest problem. More creative strategies should be designed and implemented at molecular, cellular and entire bioprocess levels to resolve the problem. In this regard, systems metabolic engineering will continue to play an important role in optimizing BC production, which aims at optimizing all the relevant bioprocesses during strain development, including the use of raw material, fermentation, and separation and purification processes (Lee and Kim, 2015). Another critical step that affects the fate of BC is a further downstream processes that physically or chemically modify BC to make biofunctionality of BC suitable for specific applications as mentioned above (Fig. 1) (Rajwade *et al*., 2015).

In summary, a versatile polymer BC can be sustainably produced from renewable resources, replacing some of fossil resource‐based materials currently being used. BC produced by fermentation can be used in diverse applications as showcased above. Once disposed after use, BC will be degraded without leaving environmentally harmful footprints, thus forming a perfect circular economy. Also, BC possesses some unique material properties compared with synthetic materials being used, and thus, it provides new functional applications beyond its environmental benefits and sustainability. Although we showcased BC as just one example here how it helps us move towards ‘sustainable production and consumption’, many other bio‐based products will contribute to this SDG in similar ways. Further research to address remaining problems, such as low production levels and further improvement of materials properties in the case of BC, will contribute to achieving sustainable consumption and production (Fig. 1).

## Conflict of interest

Authors declare that they do not have conflict of interest.
